# Primary Borderline Mucinous Tumors of the Testis: A Case Report and Literature Review

**DOI:** 10.1155/2015/863745

**Published:** 2015-03-02

**Authors:** Satoshi Funada, Toru Yoshida, Masaaki Ito, Fumihiko Kono, Takehiko Segawa

**Affiliations:** ^1^Department of Urology, Kyoto City Hospital, 1-2 Higashitakada-Cho, Mibu, Nakagyo-Ku, Kyoto 604-8845, Japan; ^2^Department of Pathology, Kyoto City Hospital, 1-2 Higashitakada-Cho, Mibu, Nakagyo-Ku, Kyoto 604-8845, Japan

## Abstract

Ovarian-type epithelial tumors of the testes and paratestes are very rare. Mucinous subtypes of such tumors are extremely rare; only 25 cases have been reported to date. Ovarian-type epithelial tumors are histologically classified into cystadenomas, borderline tumors, and carcinomas. We herein report a case involving a 60-year-old man with a primary borderline mucinous tumor of the testis. He underwent orchiectomy and has developed no recurrence for 4 years. This is the 26th report of a mucinous tumor of the testis in the literature. We also herein review the literature and discuss the etiology, prognosis, and treatment of mucinous tumors of the testes.

## 1. Introduction

Surface epithelial tumors are the most common type of ovarian neoplasms. Histologically similar tumors, namely, ovarian-type epithelial tumors, can arise from the testes and paratestes. However, these tumors are very rare; only 25 cases of the mucinous subtype have been reported [1–17]. We herein report a case of a borderline mucinous tumor of the testis and review the literature on this rare testicular neoplasm.

## 2. Case Presentation

**Figure 1:**
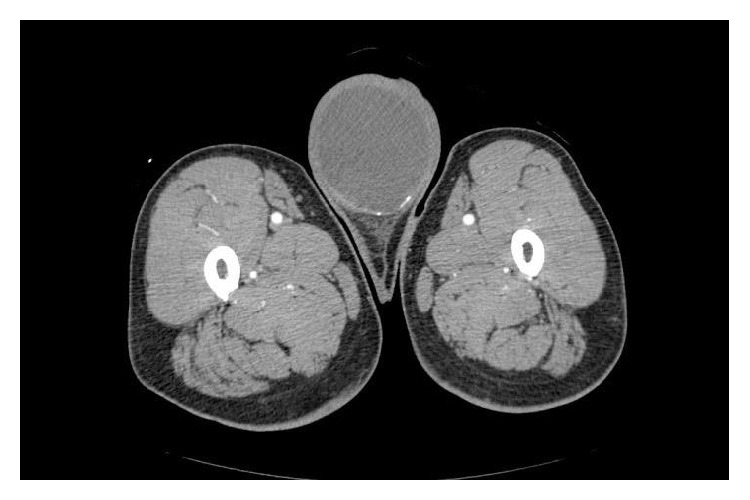
Contrast-enhanced computed tomography scan of the abdomen and pelvis showed a left testicular mass.

A 60-year-old man presented with a 5.5-year history of a painless swelling in his right testis. Physical examination findings were unremarkable with the exception of a left testicular mass. No inguinal lymphadenopathy was found on palpation. Laboratory findings showed an elevated concentration of carcinoembryonic antigen (CEA) at 12 ng/mL (normal: <5 ng/mL) with normal serum concentrations of germ cell tumor markers (lactate dehydrogenase, *β*-human chronic gonadotropin, and *α*-fetoprotein). Ultrasound examination revealed a 15 cm heterogeneous mass in the right testis. No abnormalities were seen with the exception of a left testicular mass on a contrast-enhanced computed tomography scan of the abdomen and pelvis (Figure 1).

**Figure 2:**
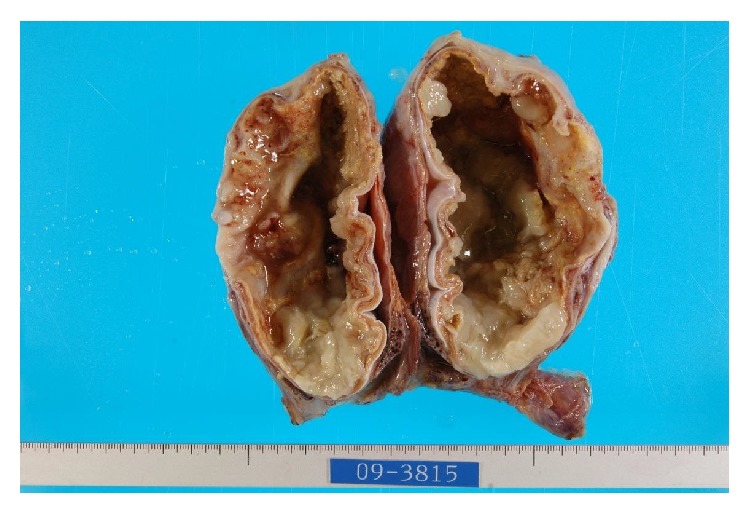
Gross examination showed a unilobular mucinous tumor measuring 14.5 × 5.0 × 12.0 cm.

The patient underwent a right inguinal orchiectomy. Gross examination revealed a unilobular mucinous tumor measuring 14.5 × 5.0 × 12.0 cm and replacing the testis (Figure 2). The outside surface of the tumor was smooth, the exudate was transparent yellow, and the inside lumen was fibrotic and gritty.

**Figure 3:**
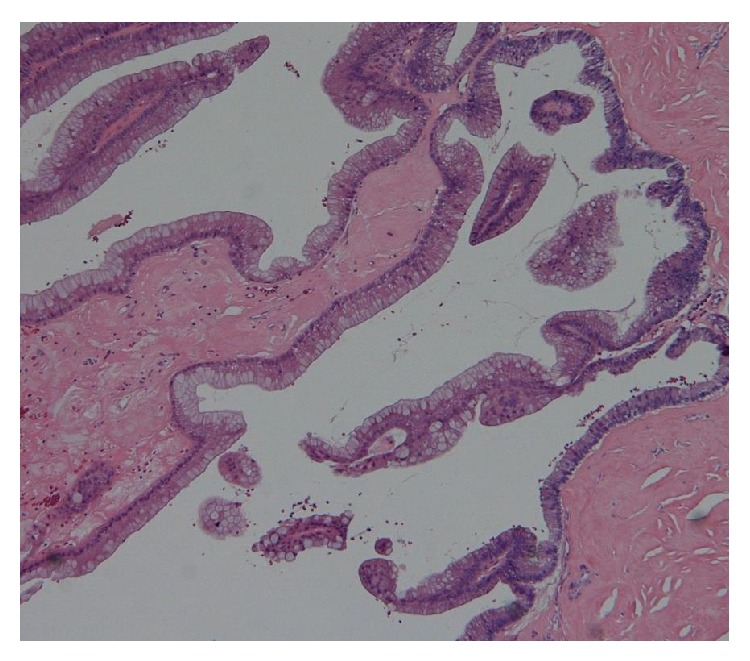
Microscopic examination showed mucinous cells lining the tumor in a papillary arrangement.

Microscopic examination showed mucinous cells lining the tumor in a papillary arrangement (Figure 3). The mucinous cells were highly atypical with no stromal invasion, qualifying as having low malignancy potential or “borderline.” The tumor had a fibrotic and hyalinized cyst wall with calcification, metaplastic ossification, and cholesterol clefts. The peritumoral testicular parenchyma was normal, and the tumor showed no evidence of teratomatous components. Immunochemical staining was positive for cytokeratin 7 and cytokeratin 20.

The patient recovered well after the surgery. He has undergone blood tests and contrast-enhanced computed tomography scans of the chest, abdomen, and pelvis every 6 months for 4 years. There has been no recurrence or elevation of the CEA concentration.

## 3. Discussion

Ovarian-type epithelial tumors of the testis are histologically identical to surface epithelial-stromal tumors of the ovary. Six histologic subtypes have been defined: serous, mucinous, endometrioid, clear, transitional (Brenner), and squamous. Similar to ovarian tumors, these tumors range from cystadenomas to borderline tumors to carcinomas. Among these subtypes, serous tumors are the most common, and borderline tumors are more common than carcinomas. In contrast, primary mucinous tumors of the testes and paratestes are very rare [15]. To the best of our knowledge, only 25 cases have been reported in the English-language literature, and the present report is the 26th. These previous reports include 17 cases of mucinous tumors in the testes and 9 cases in the paratestes. Among all 26 reported cases, 8 were cystadenomas, 13 were borderline tumors, and 5 were mucinous carcinomas.

Distinguishing borderline tumors from malignant tumors is important because of their different prognoses. Pathologically, mucinous borderline tumors show abnormal proliferation of epithelium with stratification, papillae, and filiform branching but do not invasively destroy the stroma. The 26 reported cases include 13 borderline tumors and 5 mucinous carcinomas. No recurrence or metastasis was reported for any of the borderline cases; however, 2 years after the diagnosis of mucinous carcinoma, 2 patients had died of the primary disease and 1 was alive with metastases.

The predominant (but still controversial) hypothesis of the origin of ovarian-type epithelial tumors is that they arise from metaplasia of the mesothelium of the tunica vaginalis [9, 18]. Competing hypotheses hold that they arise from Müllerian remnants in the connective tissue between the testis and epididymis or within the spermatic cord [19] or from monodermal teratomas [20].

When urologists encounter a mucinous tumor of the testis, metastatic carcinoma is a vital differential diagnosis. Metastatic mucinous tumors are more common than primary mucinous tumors in the testes. Ulbright and Young [9] reported that 53% of metastatic tumors in the testis come from carcinomas of the colon, pancreas, and stomach and that they can mimic primary mucinous tumors. Immunohistochemical studies may help to distinguish primary testicular mucinous tumors from metastatic carcinomas. Most colorectal adenocarcinomas are cytokeratin 7^−^/cytokeratin 20^+^, whereas most ovarian mucinous cancers are cytokeratin 7^+^/cytokeratin 20^+^, as seen in our case [21]. However, immunohistochemical methods give an incomplete picture; adequate radiological studies help to distinguish primary tumors from metastases. In the present case, we performed contrast-enhanced computed tomography scans of the chest, abdomen, and pelvis every 6 months for 4 years and found no evidence of tumors. Therefore, we diagnosed our case as a primary testicular neoplasm.

Because these tumors are rare, treatment experience is limited. Cystadenomas and borderline tumors have been successfully treated by radical orchiectomy only, with no recurrence or metastases. However, some mucinous carcinomas are associated with metastases and may require chemotherapy. Azuma et al. [16] and Vaughn et al. [22] reported that malignant ovarian-type epithelial tumors of the testis are sensitive to the chemotherapy used for ovarian carcinomas. They administered paclitaxel and carboplatin every 3 weeks.

In conclusion, we have reported a rare case of a borderline mucinous tumor of the testis and reviewed related reports. Although many characteristics of this tumor remain unclear, distinguishing borderline tumors from carcinomas is clearly important because the former has a good prognosis whereas the latter has a poor prognosis.
